# Degradation and ring-opening polymerization of poly(ε-caprolactone) by a novel enzyme from *Pseudomonas* sp. DS0801

**DOI:** 10.1016/j.isci.2025.114173

**Published:** 2025-11-21

**Authors:** Yao Di, Wenfei Luan, Lihua Sun, Jing Qi, Hongmei Xia, Fan Li, Niu Zhai

**Affiliations:** 1 School of Life Sciences, Northeast Normal University, Changchun 130024, China; 2 Shanghai Technical Institute of Electronics & Information, Shanghai 201411, China; 3 China Tobacco Gene Research Center, Zhengzhou Tobacco Research Institute of CNTC, Zhengzhou 450001, China

**Keywords:** biochemistry, microbiology, enzyme engineering

## Abstract

Poly(ε-caprolactone) (PCL) is a widely used synthetic polymer with significant commercial applications, and its degradation and synthesis have become the focus of considerable research. In this study, we purified a PCL-degrading enzyme, PCLase0801, produced by *Pseudomonas* sp. DS0801. The enzyme was purified to homogeneity and had a molecular weight of 30.4 kDa. It exhibited optimal activity at 40°C and pH 8.0, hydrolyzing PCL into monomers, dimers, and trimers. The enzyme was identified as a lipase and showed good tolerance to organic solvents. Additionally, PCLase0801 catalyzed the ring-opening polymerization of ε-caprolactone, producing PCL with a molecular weight of 6050 g/mol and favorable structural properties. This work provides new insights into the potential applications of PCL-degrading enzymes in PCL treatment and biosynthesis.

## Highlights

• A novel PCL-degrading enzyme was purified from the strain of *Pseudomonas* sp. DS0801
• PCLase0801 can not only degrade PCL but also reverse the synthesis of PCL
• PCLase0801 has excellent performance in temperature, pH, and organic solvent tolerance
• PCLase0801 enables rapid polymerization under mild, low-temperature conditions

## Graphical abstract

**Figure:**
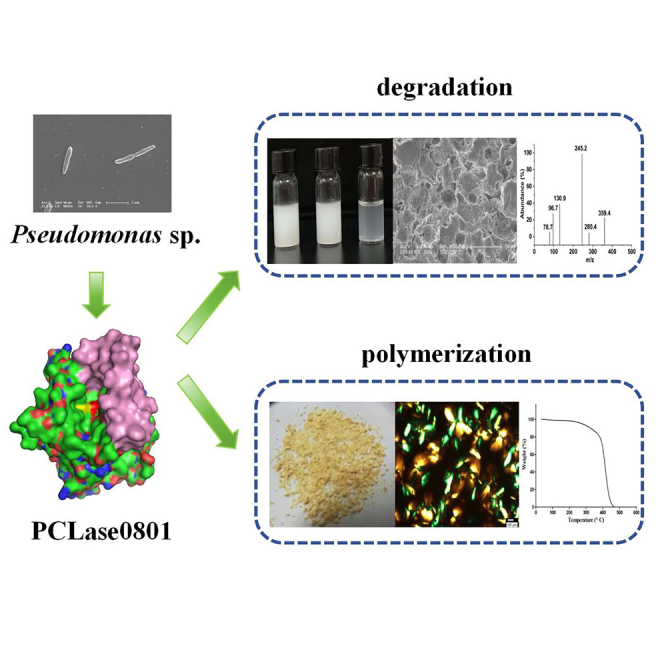

## Introduction

As environmental conservation gains prominence, there has been a growing focus on biodegradable plastics.^1^^,^^2^ Biodegradable polyesters and their composites are increasingly prominent in biomedical and ecological applications.^3^^,^^4^^,^^5^ Among these materials, poly(ε-caprolactone) (PCL) stands out as a significant aliphatic polyester due to its excellent processability, low melting point, biodegradability, and biocompatibility.^6^^,^^7^

Numerous studies have shown that the degradation of polyesters such as PCL is influenced by environmental conditions and often proceeds more slowly and less efficiently than anticipated. For instance, Lambert et al. reported that it takes 3–4 years for PCL to completely degrade in nature.^8^^,^^9^ The biodegradable polymers should degrade rapidly, in a controlled manner, and at low cost once they have fulfilled their intended purpose. Clearly, microorganisms and the polyester-degrading enzymes they produced are thought to have an advantage in this regard.^10^^,^^11^ Biocomposting utilizes microorganisms and enzymes to depolymerize polymers into monomers or oligomers, which are subsequently assimilated.^12^^,^^13^ A diverse range of PCL-degrading microorganisms, including fungi, bacteria, and actinomycetes have been isolated, characterized, and evaluated for their degradation capabilities.^14^^,^^15^ Several PCL-degrading enzymes produced by these microorganisms have been purified and characterized.^16^^,^^17^ These microorganisms and enzymes provide valuable resources for the effective degradation and biocycling of PCL. However, current research on PCL degradation primarily focuses on the evaluation of the degradation performance of PCL.^18^^,^^19^ The reported PCL-degrading enzymes are insufficient in terms of diversity and systematic characterization. In addition, the biodegradation of PCL catalyzed by enzymes is often limited by various environmental conditions. Factors such as pH, temperature, anaerobic or aerobic environment, light condition, and wet saturated environment will affect the degradation process of PCL.^9^ Therefore, it is of great significance to explore more PCL-degrading enzymes and study their characteristics, which is the premise of regulating PCL degradation.

Due to PCL’s superior properties, its synthesis and production have also attracted significant research attention. As we all know, PCL is a synthetic polyester, and its initial production method is chemical synthesis. Chemical polymerization, which typically requires high temperatures (150°C–280°C) and involves toxic residues from organometallic catalysts, can limit the application of PCL in specific fields.^20^ In recent years, enzymatic synthesis of PCL has received much attention because it operates under mild conditions and eliminates the introduction of metal ions.^20^^,^^21^^,^^22^ If PCL-degrading enzymes can also catalyze the reverse synthesis of PCL, an ideal recycling system could be established.

In previous research, we isolated a strain capable of effectively degrading PCL from activated sludge, which was identified as *Pseudomonas* sp. DS0801. This strain can be induced to produce PCL-degrading enzymes when cultured on a medium containing PCL as the sole carbon source. In the current study, we purified a PCL-degrading enzyme from this strain and examined its catalytic properties and degradation mechanisms. Furthermore, given that certain hydrolases capable of cleaving ester bonds, such as lipases, can also catalyze esterification and polymerization reactions, we further investigated whether the PCL-degrading enzyme described in this study could facilitate the ring-opening polymerization of polyesters, thereby enabling the biosynthesis of PCL through reverse synthetic pathways. The enzyme’s remarkable degradation and reverse synthesis capabilities suggest its substantial potential for practical applications.

## Results and discussion

### Purification and PCL degradability of the purified enzyme

**Figure 1:**
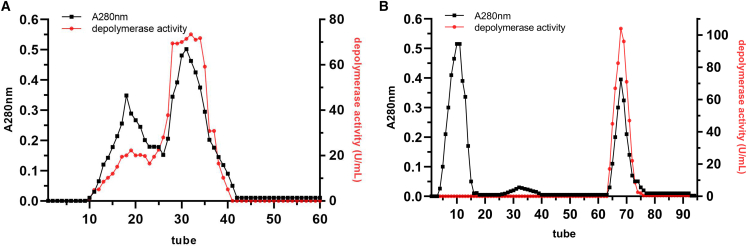
Elution profile of PCL-degrading enzyme (A) Elution profile obtained via DEAE Sepharose Fast Flow column (1.6 × 30 cm). (B) Elution profile obtained via Phenyl Sepharose 6 Fast Flow column (1.6 × 30 cm). The sample was eluted from the DEAE Sepharose Fast Flow column using a linear NaCl gradient from 0 to 1.0 M at a flow rate of 0.5 mL/min. Active fractions were then applied to the Phenyl Sepharose 6 Fast Flow column and eluted with a decreasing linear ammonium sulfate gradient from 0.8 M to 0 at the same flow rate. Black squares represent absorbance at 280 nm, and red dots represent depolymerase activity.

**Figure 2:**
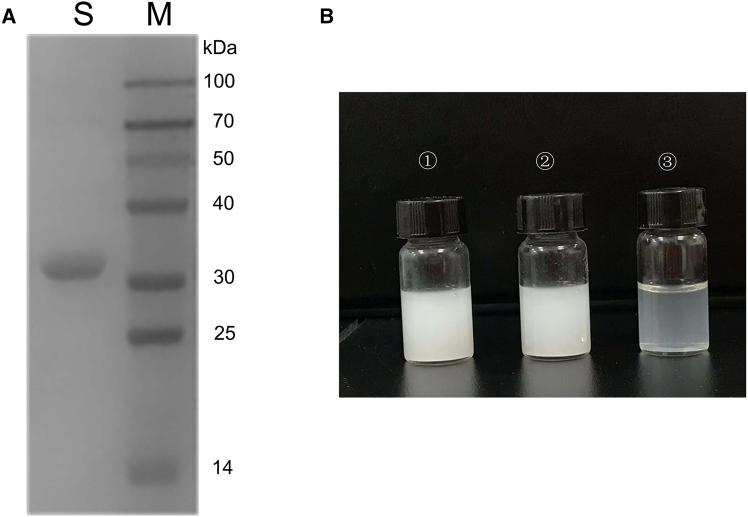
SDS-PAGE and hydrolytic activity of the purified PCL-degrading enzyme (A) SDS-PAGE. Lane M, molecular weight marker; Lane S, purified PCL-degrading enzyme. (B) Hydrolytic activity of the purified PCL-degrading enzyme. ①, PCL emulsion incubated with phosphate buffer; ②, PCL emulsion incubated with heat-inactivated PCLase0801; ③, PCL emulsion incubated with active PCLase0801.

The PCL-degrading enzyme was purified to homogeneity from the culture supernatant of the *Pseudomonas* sp. DS0801 strain. The elution profiles are shown in Figure 1. The purified enzyme, designated PCLase0801, has a molecular mass of 30.4 kDa, as determined by SDS-PAGE analysis (Figure 2A). The PCL emulsion incubated with purified PCLase0801 was significantly clarified. In contrast, no decrease in turbidity was observed when PCL emulsion was mixed with phosphate buffer or a heat-inactivated enzyme. These results indicate that the purified enzyme can effectively degrade PCL (Figure 2B).

### Enzymatic properties of PCL-degrading enzyme

#### Impact of temperature and pH on the degradation activity of PCLase0801

**Figure 3:**
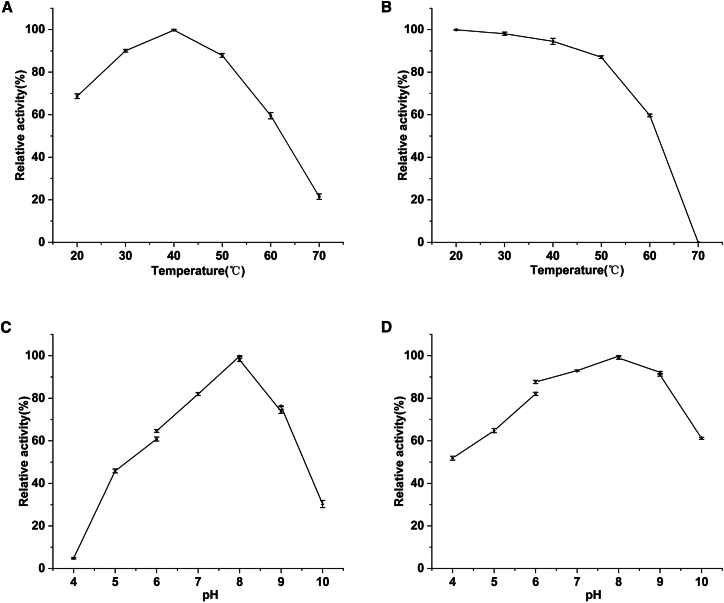
Effects of temperature and pH on the PCL-degrading activity of PCLase0801 (A) Temperature dependence of enzymatic activity. (B) Thermostability of the purified enzyme. (C) pH dependence of enzymatic activity. (D) pH stability of the purified enzyme. Buffers used: citrate buffer (pH 4.0–6.0), Na_2_HPO_4_-NaH_2_PO_4_ buffer (pH 6.0–8.0), Tris-HCl buffer (pH 8.0–9.0), Gly-NaOH buffer (pH 9.0–10.0).

Temperature and pH significantly influence the catalytic activity and stability of enzymes. The temperature range for the analysis of PCL-degrading activity was set from 20°C to 70°C. Purified PCLase0801 exhibited maximum degradation activity at 40°C, as shown in Figure 3A. Notably, enzyme activity decreased significantly at temperatures above 60°C. In comparison with previous studies, many PCL-degrading enzymes derived from fungi and bacteria exhibit maximum activity around 50°C.^23^^,^^24^^,^^25^ PCLase0801, however, achieves its maximum reaction rate at a relatively low temperature, making it more suitable for operation at ambient temperature and helping to reduce energy consumption. Additionally, the enzyme maintained stability at temperatures up to 50°C for 4 h; however, stability declined sharply when the temperature exceeded 60°C (Figure 3B). The optimal pH for PCLase0801 activity was determined to be 8.0, with relatively high enzyme activity maintained between pH 7.0 and 9.0 (Figure 3C). PCLase0801 retained over 80% of its enzymatic activity across pH conditions ranging from 6.0 to 9.0 after 24 h of incubation. However, it became unstable when the pH dropped below 5.0 or exceeded 10.0 (Figure 3D). Several PCL-degrading enzymes, as reported by B. Ruiz and M. Amin et al., also exhibited both stability and notable activity within the pH range of 7.0–9.0.^26^^,^^27^ While some enzymes show a preference for acidic environments,^24^ those with optimal pH in the neutral range have a broader range of applications.

#### Impact of metal ions and chemicals on the degradation activity of PCLase0801

Enzyme activity is influenced by both the concentration and type of metal ions present in the reaction. Metal ions may interact with the enzyme’s active site or potentially affect its conformation and stability through electrostatic interactions with amino acids.^28^^,^^29^ Table 1 summarizes the effects of metal ions on the degradation activity of PCLase0801. These findings show that K^+^ and Mn^2+^ promote PCLase0801 activity, while Cu^2+^, 10 mM Fe^2+^, and 10 mM Fe^3+^ significantly inhibited it.

**Table 1:** Effect of metal ions on the PCL-degrading activity of PCLase0801

Metal ions	Residual activity (%)	
	1 mM	10 mM
KCl	102 ± 0.9	159.4 ± 1.9
ZnCl_2_	70.1 ± 0.7	51.8 ± 5.2
CaCl_2_	77.4 ± 1.7	47.1 ± 3.8
CoCl_2_	90.0 ± 3.5	–
FeCl_2_	125.3 ± 6.2	35.3 ± 4.1
FeCl_3_	88.8 ± 4.3	10.4 ± 2.9
CuSO_4_	0	–
MnSO_4_	107.1 ± 1.4	120.4 ± 4.3

– Means that the effect of the metal ion could not be detected for the precipitate.

Table 2 summarizes the impact of chemicals on the PCL-degrading activity of PCLase0801. Methanol, ethanol, and glycerol at 1% concentration had minimal effects on enzyme activity. Even at higher concentrations of organic solvents, the enzyme remained highly active, suggesting that PCLase0801 exhibits tolerance to organic solvents. The PCL-degrading enzyme reported by P. Anbu et al. also shows high resistance to organic solvents, maintaining strong catalytic activity in such environments.^30^ Similarly, PCLase0801 demonstrates comparable solvent resistance and holds potential for applications in high-organic-solvent environments. This stability may be due to its open conformation, which prevents obstruction of the active site and allows for a flexible structure.^27^ Consequently, the stability of PCLase0801 in organic solvents makes it a promising candidate for reactions involving these solvents.

**Table 2:** Effect of chemicals on the PCL-degrading activity of PCLase0801

Organic solvent	Residual activity (%)	
	1% (V/V) or 1 mM	10% (V/V) or 10 mM
Ethanol^a^	95.6 ± 1.4	68.2 ± 2.1
Methanol^a^	96.2 ± 0.5	82.3 ± 3.1
Glycerol^a^	97.9 ± 2.2	78.4 ± 0.8
Triton X-100^a^	13.8 ± 1.9	0
Tween-80^a^	12.7 ± 1.2	0
PMSF^b^	95.7 ± 2.5	76.3 ± 3.8
EDTA^b^	93.8 ± 0.6	64.0 ± 1.8
SDS^b^	16.2 ± 2.0	0

a Means final concentrations of 1% (v/v) and 10% (v/v).

b Means final concentrations of 1 mM or 10 mM.

The presence of SDS, Triton X-100, or Tween-80 resulted in significant inhibition of enzyme activity. As a metal ion chelator, 10 mM EDTA inhibited enzyme activity, suggesting that metal ions are essential for the function of PCLase0801. PMSF, a serine protease inhibitor, also inhibited the enzyme, indicating the presence of a critical serine residue within the enzyme’s catalytic center.

#### Substrate specificity of PCLase0801

Several representative substrates were used to evaluate the activity of PCLase0801. The results revealed that PCLase0801 exhibited significant degradability toward PCL but showed no activity against PHB or PLA. This can be attributed to the intrinsic properties of the polyester. The chemical structure, molecular weight, and degree of polymerization of various polyesters influence their chain length and molecular complexity, thereby affecting the enzyme’s ability to degrade different polyesters.^28^ Additionally, PCLase0801 demonstrated the capacity to degrade tributyrin, olive oil, and *p*-nitrophenyl esters (C2 to C16), with increased activity observed on long-chain substrates (Figure S1), suggesting that PCLase0801 may function as a lipase. Subsequently, MALDI-TOF-MS analysis and alignment were performed on purified PCLase0801. The alignment results showed that PCLase0801 shares 99.99% sequence similarity with a lipase derived from *Pseudomonas aeruginosa*. Based on homologous sequence analysis, the PCLase0801 gene was successfully cloned, and its nucleotide sequence has been deposited in GenBank (GenBank: PX234425). Sequence analysis revealed that PCLase0801 shares 98.39% sequence identity with triacylglycerol lipase from *Pseudomonas aeruginosa*. To date, no studies have reported the function of this triacylglycerol lipase in PCL degradation.

#### Hydrolysis products analysis of PCLase0801

**Figure 4:**
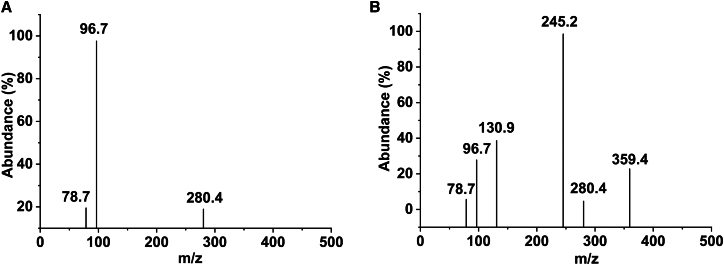
Mass spectrometry analysis of PCLase0801 degradation products (A) Control group. (B) PCLase0801. Compared with the control, monomers (m/z, 130.9), dimers (m/z, 245.2), trimers (m/z, 359.4) were detected in the enzyme-treated sample. Other peaks correspond to matrix signals or unrelated reaction components.

Mass spectrometry (MS) was employed to identify the breakdown products of PCL after treatment with PCLase0801. The analysis revealed the presence of monomers, dimers, and trimers in the treated samples, compared with the control group (Figure 4). These findings suggest that the catalytic active site of PCLase0801 interacts with extended substrates composed of multiple PCL units, cleaving the ester bonds within.

### Degradation process and SEM analysis of the films

**Figure 5:**
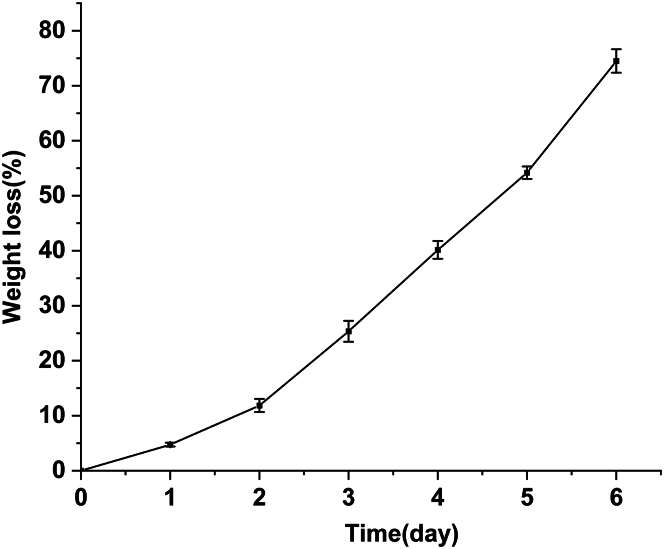
Weight loss of PCL films during enzymatic degradation PCL films (10 × 10 × 0.2 mm) were incubated with PCLase0801 (0.2 mg/mL). The enzyme solution was refreshed daily, and the films were collected and vacuum dried every 24 h.

**Figure 6:**
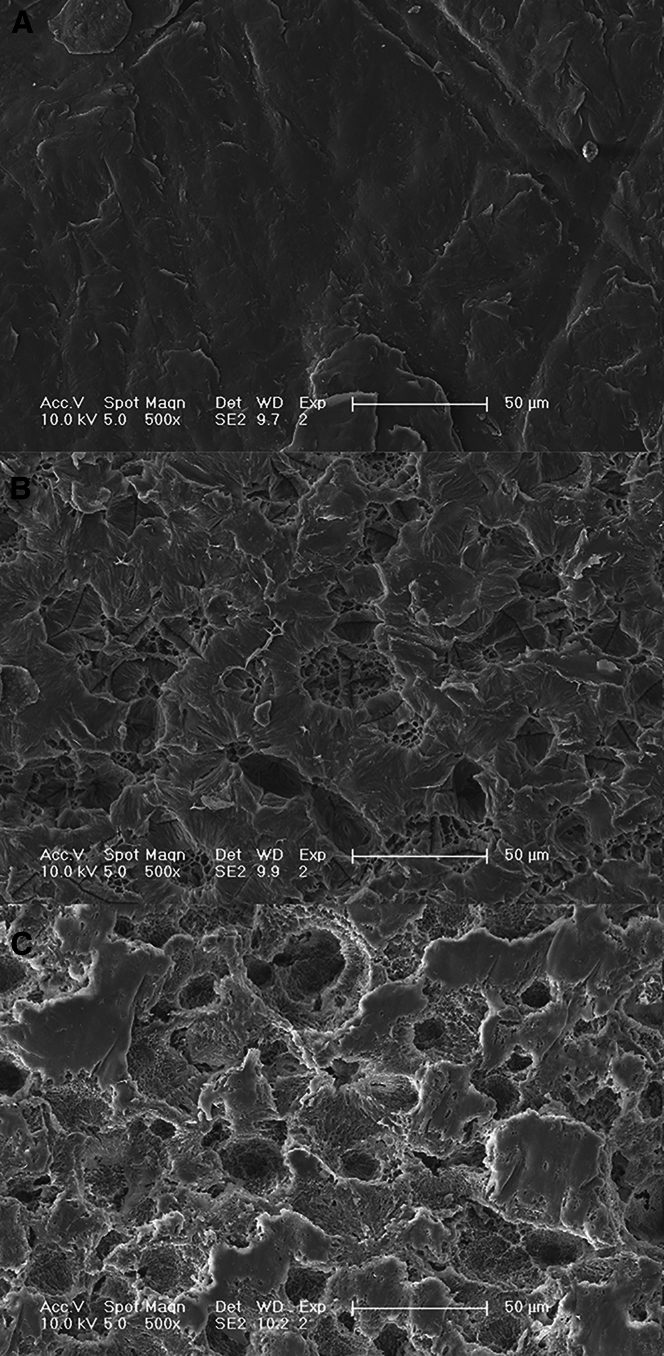
SEM micrographs of PCL films (A) Undegraded PCL. (B) PCL films degraded by PCLase0801 for 72 h. (C) PCL films degraded by PCLase0801 for 120 h. Images were obtained at an acceleration voltage of 15 kV.

The degradation of PCL films by PCLase0801 resulted in a significant weight loss, as illustrated in Figure 5. This process occurred in two distinct stages: an initial slow stage (0–2 days) followed by a rapid stage (2–6 days) (Figure 5). After 6 days of incubation, the total weight loss exceeded 73%. The observed increase in the degradation rate may be attributed to the increase in the surface area accessible to the enzyme as the degradation progressed. Additionally, the degradation of the molecular chains exposed more binding sites for enzyme interactions. Figure 6 shows scanning electron microscope (SEM) images of the PCL films after degradation by PCLase0801. Prior to enzymatic hydrolysis, the PCL film surface appeared smooth (Figure 6A). As degradation progressed, the surface sustained damage, leading to increased roughness and crack depth (Figure 6C). Shi et al. demonstrated that various enzyme types exhibit distinct degradation behaviors on PCL. For instance, during cutinase degradation, PCL undergoes layer-by-layer degradation, whereas lipase degradation may result in the formation of large pores on the PCL membrane surface. Our findings support this conclusion.^16^^,^^31^ Enzymatic hydrolysis of polyesters generally proceeds via surface erosion, as extracellular enzymes act only upon contact with the polymer surface. Recent studies have shown that embedding enzymes within the polymer matrix can create internal enzyme-polymer interfaces and markedly accelerate degradation.^32^^,^^33^ This novel strategy may enable PCL-degrading enzymes to achieve more efficient degradation of solid polycaprolactone waste.

### Enzymatic polymerization of ε-caprolactone by PCLase0801

Some lipases have been shown to catalyze the synthesis of polyesters in non-aqueous media.^20^^,^^21^^,^^34^ However, the ability of PCL-degrading enzymes to synthesize polyester through the reverse reaction of hydrolysis has not been reported. Our results demonstrate that PCLase0801 catalyzes the ring-opening polymerization of ε-caprolactone in an n-hexane system, yielding PCL with a conversion rate of 72.75%. Many enzymes that catalyze ring-opening polymerization also possess polycondensation activity. Polycondensation is a commonly employed method for polymer synthesis. To further explore the polymerization ability of PCLase0801, we investigated its capacity to catalyze polycondensation. The polycondensation of diols and diacids with different chain lengths showed that PCLase0801 could catalyze nine types of polymerization reactions (Table 3), demonstrating broader enzymatic polymerization capability than previously reported.^34^^,^^35^

**Table 3:** PCLase0801 catalyzed polymerization between diacids and diols

	Yield (%)		
	1,4-butanediol	1,6-hexanediol	1,8-octanediol
Adipic acid	15.34	36.40	26.10
Suberic acid	18.54	46.59	56.68
Sebacic acid	7.15	27.21	16.59

#### Optimization of reaction conditions

To increase the conversion of monomers in the catalytic synthesis of PCL, we optimized key parameters in the enzymatic polymerization process. The optimal reaction system for ε-caprolactone ring-opening polymerization catalyzed by PCLase0801 consisted of 25 mg of enzyme powder, a temperature set at 45°C, a duration of 8 h, hexane as the solvent, and a water activity of 0.11 (details are provided in the Supplementary Materials, Figure S2–S6 and Tables S1 and S2). The reaction system for ε-caprolactone ring-opening polymerization reported by Li and Ma et al. was at 80°C–90°C and the reaction time was 72 h,^34^^,^^35^ other lipases even require more than 10 days.^20^ Compared with other reported enzyme catalysts, PCLase0801 is quite efficient. This system offers the significant advantages of reduced reaction time and lower temperature, making it suitable for industrial applications.

#### Characterization of the structure of polymerized PCL

The product obtained from the ring-opening polymerization of ε-caprolactone is a yellow, waxy solid, as shown in Figure S6. It exhibits high fluidity upon melting and rapid solidification upon cooling, indicating excellent processability and moldability.

**Figure 7:**
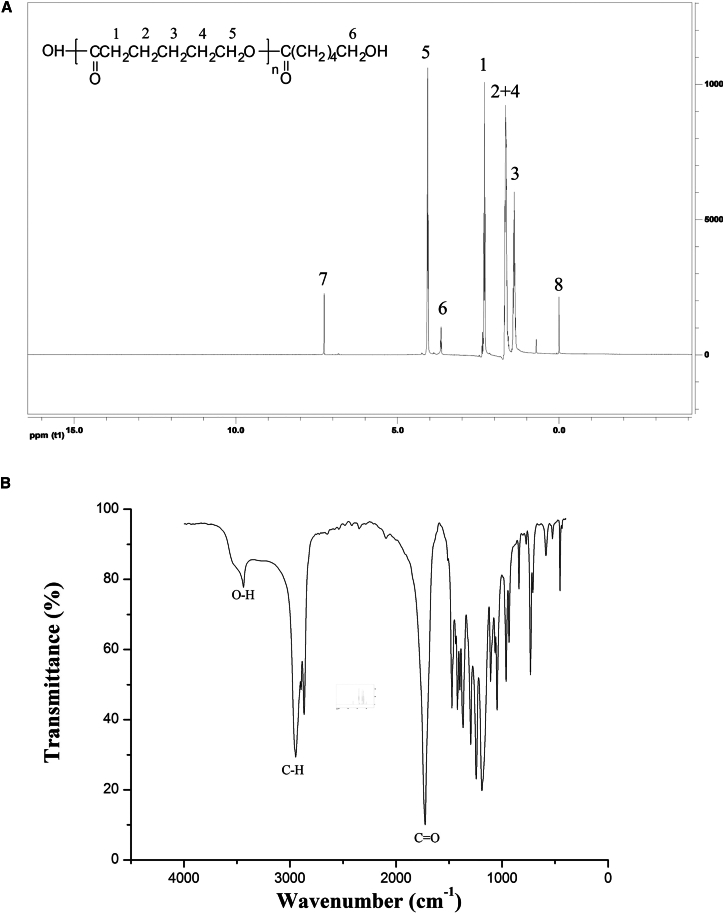
Characterization of the structure of polymerized PCL (A) ^1^H NMR spectrum. (B) FT-IR spectrum. The ^1^H NMR spectrum was recorded in chloroform-d on a Bruker-400 spectrometer with tetramethylsilane (TMS) as the internal standard.

The ^1^H NMR spectrum, shown in Figure 7A, confirmed the structure of the product. Peaks 1–5 correspond to the characteristic splitting of hydrogen atoms in the repeating units of the PCL chemical structure. Peak 6 corresponds to the characteristic splitting of the two hydrogen atoms on the terminal carbon atom of PCL, indicating that the terminal carbon is bonded to a hydroxyl group. Peak 7 represents the hydrogen atom in the deuterated chloroform solvent used in the analysis, while peak 8 corresponds to the hydrogen atom of the internal standard tetramethylsilane. The Fourier transform infrared (FT-IR) spectrum of the synthesized product is shown in Figure 7B. FT-IR analysis of PCLase0801 revealed a C=O stretching vibration peak at 1731.2 cm^−1^. The band near 2936.3 cm^−1^ was attributed to the stretching vibrations of C–H bonds. The peak at 3434.9 cm^−1^ confirmed the presence of a hydroxyl group, further verifying the formation of a linear polymer chain.^10^ These three characteristic peaks are consistent with the chemical structure of PCL.

#### Properties of the polymerized PCL

The polymerized PCL exhibited a number-average molecular weight (Mn) of 6,050 g/mol and a weight-average molecular weight (Mw) of 11,074 g/mol. The polydispersity index was calculated to be 1.7024, indicating a relatively uniform molecular weight distribution. These results suggest that PCLase0801 is well suited for producing low-molecular-weight PCL, making it a promising candidate for applications such as drug delivery systems or as a soft segment in polyurethane formulations.^36^ PCL is widely used in biomedicine due to its favorable biocompatibility. In contrast, traditional chemical synthesis methods often require metal catalysts, which can be harmful even in trace amounts.^37^ Enzymatic polymerization, however, avoids metal toxicity, thus enhancing its suitability for medical applications.

**Figure 8:**
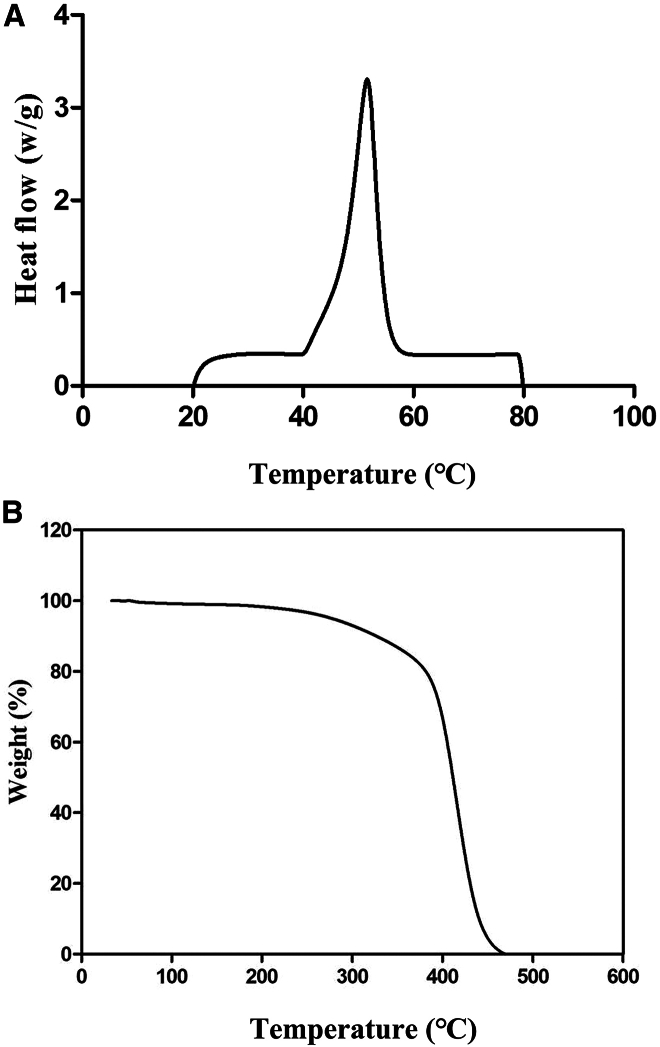
Characterization of the properties of polymerized PCL (A) DSC curves. (B) TGA curves. For DSC analysis, a sample (6 mg) was heated from 20°C to 80°C at 10 °C/min under nitrogen. For TGA analysis, a sample (8 mg) was heated from 20°C to 600°C at 10°C/min under nitrogen.

The results of the differential scanning calorimetry (DSC) test are presented in Figure 8A. The polymerized PCL synthesized using PCLase0801 exhibited a melting point of 51.62°C and a melting enthalpy (ΔHm) of 111.3 J/g. The crystallinity of the polymerized PCL was calculated to be 84%. In comparison, Gumel et al. employed ultrasound-assisted lipase to catalyze the ring-opening polymerization of ε-caprolactone, resulting in PCL with a crystallinity of 61%. PCL prepared through traditional catalytic methods exhibited a significantly lower crystallinity of only 28%.^38^ Our polymerization product has a higher crystallinity than previous research. Higher crystallinity in polymers is associated with a greater proportion of crystalline regions and a more compact crystal arrangement, which contribute to enhanced mechanical strength and hardness.

Thermogravimetric analysis (TGA) is widely used to assess material thermal stability. The analysis showed that PCL produced by PCLase0801 exhibited an initial decomposition temperature of 250°C, with the rate of thermal weight loss increasing most rapidly at approximately 350°C. Complete thermal degradation occurred at 460°C (Figure 8B). Ma et al.^35^ utilized lipase (CALB) from *Candida antarctica* to catalyze the polymerization of ε-caprolactone, with the product showing an initial thermal decomposition temperature of 280°C, a peak weight loss rate at approximately 340°C, and complete thermal degradation at 400°C. Similarly, Gumel et al. used ultrasound-assisted lipase to synthesize PCL, and the initial thermal decomposition temperature was recorded at 330°C, with the highest weight loss rate at approximately 380°C and complete degradation at 464°C.^38^ Our PCL product demonstrates similar thermal decomposition characteristics and exhibits strong thermal stability compared with these findings.

**Figure 9:**
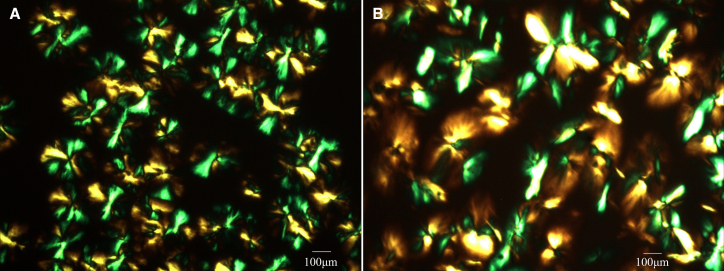
Polarized optical micrographs (200×) of isothermal crystallization of poly(ε-caprolactone) (A) Commercial PCL. (B) PCL synthesized by PCLase0801. Samples were heated to 90°C at 30°C/min, equilibrated for 3 min, and then cooled to 40°C.

Additionally, we examined the crystal morphology of the enzymatically synthesized PCL product using a polarized light microscope. The polarized light micrographs confirmed that the enzymatic synthesis product has crystallization capability, with spherulite sizes similar to those observed in commercial PCL (Figure 9).

### Mechanistic insights into PCLase0801function

#### Hydrolytic action of PCLase0801

PCL is an artificially synthesized polyester that does not occur in nature. Current studies suggest that the degradation enzymes are not specifically evolved for PCL’s structure, but are likely esterases, lipases, or cutinases with broad substrate specificity that can bind to the long chain of PCL.^10^ Due to the diversity of these enzymes, the relationship between the enzyme structure and its ability to degrade PCL remains unclear. More PCL-degrading enzymes need to be characterized to better understand the mechanism by which synthetic compounds can be hydrolyzed by enzymes. This is of great significance for the degradation of existing materials and the development of new biodegradable materials.

**Figure 10:**
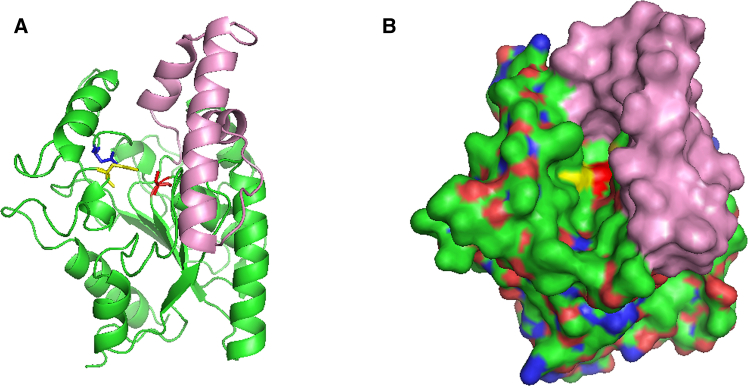
Modeled structure of *Pseudomonas* sp. PCLase0801 (A) Stereo view showing the “core” domain and “cap” domain. (B) Surface representation of the enzyme showing the active-site pocket. The catalytic triad residues Ser108, Asp255, and His277 are shown as sticks in red, yellow, and blue, respectively. The cap domain is marked in purple.

Based on substrate specificity assays and sequence alignment, PCLase0801 was identified as a lipase. Structural modeling of PCLase0801 revealed that the structure of PCLase0801 consists of a “core” domain (residues 26–134 and 190–311) and a “cap” domain (residues 135–189). The core domain exhibits the characteristic features of the α/β hydrolase fold topology, while the cap domain contains four α-helices that form the active-site cleft (Figure 10). The catalytic triad residues Ser108, Asp255, and His277 are located within the α/β hydrolase fold (Figure 10). Based on the reports on the mechanism of lipase action and the structural basis of PCLase0801, it is speculated that Ser108 assisted by His227 catalyzes the formation and breakdown of the tetrahedral intermediate and the acyl-enzyme complex through nucleophilic attack and proton transfer, ultimately leading to PCL degradation and the release of free carboxylic acids.

#### Polymerization action of PCLase0801

PCL synthesis methods can be classified into two categories based on the type of catalyst used: chemical polymerization and enzymatic polymerization. Chemical polymerization involves toxic residues from organometallic catalysts, limiting the application of PCL in certain fields. In contrast, enzymatic polymerization operates under milder conditions and avoids the introduction of metal ions.^20^^,^^21^ This is particularly relevant for biomedical applications, highlighting the potential of using safe natural catalysts, such as enzymes, in the ring-opening polymerization of ε-caprolactone.

Several enzymes have been reported to catalyze polyester synthesis in organic solvents.^34^^,^^35^ Our findings demonstrate that PCLase0801 likewise possesses this catalytic capability. PCLase0801 first forms a complex with the lactone and catalyzes ring opening to generate an acyl-enzyme intermediate. A nucleophilic attack by the initiator then yields a ω-hydroxycarboxylic acid, whose terminal hydroxyl group subsequently attacks the active monomer, extending the polymer chain by one unit and completing the PCL synthesis process. A crucial step in this process is the nucleophilic attack of the serine residue at the active site on the carbonyl group of the lactone, leading to the formation of an acyl-enzyme intermediate. Structural analysis and sequence alignment revealed that the key catalytic site of PCLase0801 is Ser108.

### Limitations of the study

Although our findings are valuable, this study also has certain limitations. (1) We attempted the heterologous expression of PCLase0801, but were unsuccessful in a prokaryotic expression system. Consequently, this work focuses on a mechanistic analysis based on the enzyme’s sequence rather than experimental validation using mutants. (2) During enzymatic catalysis, both catalytic efficiency and cost greatly influence the enzyme’s application potential. Therefore, approaches such as enzyme immobilization should be considered to improve enzyme utilization. Our present study provides a solid foundation; however, further efforts are required to optimize the immobilization and recovery processes. These optimization strategies will be explored in future research to enhance the enzyme’s industrial applicability.

## Resource availability

### Lead contact

For additional information, data, or resource requests, please contact the lead contact, Fan Li (lif885@nenu.edu.cn).

### Materials availability

No new unique reagents were generated in this study.

### Data and code availability

Data: The nucleotide sequence of PCLase0801 has been deposited in GenBank (GenBank: PX234425).

Code: This study does not report original code.

Additional information requests: Additional information for reanalyzing the data reported in this paper is available from the lead contact upon request.

## Acknowledgments

This research received funding from the 10.13039/501100001809National Natural Science Foundation of China (No. 31870048).

## Author contributions

Investigation, Y.D., F.L., N.Z., W.L., and J.Q.; writing-original draft, Y.D.; data curation, W.L. and L.S.; conceptualization, H.X., F.L., and N.Z.; funding acquisition, F.L.; resources, F.L.; supervision, F.L. and N.Z.; writing-review & editing, F.L. and N.Z.

## Declaration of interests

The authors declare no competing of interests.

## STAR★Methods

### Key resources table

**Table:** 

REAGENT or RESOURCE	SOURCE	IDENTIFIER
**Bacterial and virus strains**		

*Pseudomonas* sp. DS0801	Laboratory stock	DS0801

**Chemicals, peptides, and recombinant proteins**		

Phenyl Sepharose 6 FF	GE Healthcare Bio-Sciences AB	Cat#17097303
DEAE Sepharose FF	GE Healthcare Bio-Sciences AB	Cat#17070901
PCL granules	Solvay Interox LTD	Cat#CAPA6800
ε-caprolactone	Aladdin Bio-Chem Technology Co., LTD	Lot#F1518133
Plysurf A210G	Daiichi Pharmaceutical Co., LTD	Lot#9711c2
PHB	Sigma-Aldrich	Cat#916358
PLA	Sigma-Aldrich	Cat#38534
Tributyrin	Solarbio	Cat#G9420
Olive oil	Solarbio	Cat#O8322
Dichloromethane	Aladdin Bio-Chem Technology Co., LTD	Lot#L1607004

**Critical commercial assays**		

BCA Plus Protein Assay Kit	Beyotime Biotechnology	Cat#P0009

**Deposited data**		

The nucleotide sequence of PCLase0801	This paper	GenBank: PX234425

**Software and algorithms**		

PyMOL	Schrodinger, LLC	https://github.com/schrodinger/pymol-open-source
Origin 2021	OriginLab Corporation	–
SWISS-MODEL	Expasy web server	https://swissmodel.expasy.org/

**Other**		

4700 Proteomics Analyzer	ABI	–
GPS Explorer™	ABI	v3.5

### Experimental model and study participant details

*Pseudomonas* sp. DS0801 was isolated and preserved in our laboratory. The strain was screened and cultivated using PCL as the sole carbon source. The composition of the medium is described in the STAR Methods section.

### Method details

#### Cultivation medium

A total of 0.1 g of PCL granules was dissolved in a small amount of chloroform. Subsequently, 60 mL of distilled water and 0.04 g of emulsifier Plysurf A210G were added. The solution was sonicated (300 W, pulse on 7 s, pulse off 9 s, 60 cycles) until a homogeneous, milky white emulsion was formed. The PCL emulsion was obtained by stirring and heating at 70°C for 2 h to remove chloroform using a rotary evaporator. To prepare the PCL-emulsified medium, the PCL emulsion was mixed with 0.554% KH_2_PO_4_, 1.194% Na_2_HPO_4_·12H_2_O, 0.0005% CaCl_2_·2H_2_O, 0.05% MgSO_4_·7H_2_O, and 0.1% NH_4_Cl.^39^

#### Purification of PCL-degrading enzyme

*Pseudomonas* sp. DS0801 was cultured in PCL-emulsified medium for two days at 37°C. The culture supernatant was collected by centrifugation at 14,000 rpm for 30 min and concentrated via ultrafiltration using an 8 kDa molecular weight cutoff membrane (Millipore, USA). The concentrated solution was freeze-dried and then re-dissolved in a 20 mM phosphate buffer (pH 8.0). After dialysis, the sample was loaded onto DEAE Sepharose FF columns and eluted using a linear NaCl gradient from 0 to 1.0 M. The active fractions were collected, diluted to the required ammonium sulfate concentration, and then loaded onto Phenyl Sepharose 6 FF columns. Elution was performed using a decreasing linear gradient of ammonium sulfate, starting from 0.8 M to 0 M. The eluate fractions were collected, and the activity was subsequently evaluated. All purification steps were performed at 4°C.

#### Assessment of enzymatic activity

A substrate solution containing PCL at a concentration of 0.1% (w/v) was emulsified with Plysurf A210G. To assess enzyme activity, the enzyme was incubated with the substrate solution at 40°C for 20 min. The final enzyme concentration was approximately 0.1 mg/mL. The decrease in turbidity of the reaction mixture was measured using a spectrophotometer. Enzyme activity was defined as one unit (U), representing the amount of enzyme required to reduce the absorbance by 0.001 per min at 650 nm.^40^ In the one factor at a time (OFAT) test, all variables were measured according to standard enzyme activity methods, except for the single factor being varied.

#### Impact of temperature and pH on the degradation activity of the purified enzyme

The enzyme’s activity was assessed over a temperature range of 20°C–70°C to determine the optimal temperature. Its optimal pH was identified by measuring its activity in 0.1 M buffers at various pH levels: pH 4.0–6.0 (citrate buffer), pH 6.0–8.0 (Na_2_HPO_4_-NaH_2_PO_4_ buffer), pH 8.0–9.0 (Tris-HCl buffer), and pH 9.0–10.0 (Gly-NaOH buffer). To evaluate the enzyme’s thermostability and pH stability, it was incubated at temperatures ranging from 20°C to 70°C for 4 h or at pH values from 3 to 12 at 4°C for one day before the enzyme activity was determined.

#### Impact of metal ions and chemicals on the degradation activity of the purified enzyme

Several metal ions were introduced at concentrations of 1 mM or 10 mM to evaluate their impact on the enzyme’s degradation activity. These ions included K^+^, Zn^2+^, Ca^2+^, Co^2+^, Fe^2+^, Fe^3+^, Cu^2+^, and Mn^2+^. Additionally, the effects of chemicals such as ethanol, methanol, glycerol, Tween-80, and Triton X-100 was assessed at concentrations of 1% (v/v) or 10% (v/v). Ethylenediaminetetraacetic acid (EDTA), phenylmethanesulfonyl fluoride (PMSF), and sodium dodecyl sulfate (SDS) were also tested at concentrations of 1 mM or 10 mM.

#### Substrate specificity of the purified PCL-degrading enzyme

To assess the substrate specificity of the enzyme, a range of substrates was tested. The extent of substrate degradation was evaluated through turbidimetry or titration.^11^^,^^41^ The enzyme was added to emulsions of PCL, PHB and PLA to evaluate its degradation activity against various polyesters. The enzyme’s ability to degrade PHB was assessed by measuring the reduction in turbidity of the reaction mixture. PLA degradation was evaluated by detecting the production of lactic acid.^42^ The degradation of *p*-nitrophenyl (*p*-NP) esters by the enzyme was carried out at 40°C for 10 min in a final volume of 1 mL containing 900 μL of 2 mM *p*-NP substrates and 100 μL of enzyme solution. The enzyme activity on tributyrin and olive oil was determined by acid-base titration after incubation with each substrate at 40°C for 10 min.

#### Hydrolysis products analysis of the purified PCL-degrading enzyme

The enzyme and substrate solution were mixed and incubated at 40°C for 30 min. The supernatant was then analyzed by mass spectrometry (MS) using a Quattro Premier XE system equipped with capillary voltage of 3.0 kV, cone voltage of 20 V, and source temperature of 110°C.^10^ A control sample was prepared using the same substrate with an inactivated enzyme.

#### Mass spectrometry analysis of the purified PCL-degrading enzyme

The protein band corresponding to the enzyme was excised from the SDS‒PAGE gel. After washing, the gel was decolorized using 50% (v/v) ACN/25 mM ammonium bicarbonate (pH 8.0) and then dehydrated with ACN. After digestion with trypsin (0.1 mg/mL) at 37°C for 16 h, the sample was analyzed via MALDI-TOF-MS using a 4,700 Proteomics Analyzer (Tianjin Biotechnology Inc., China). The MASCOT Peptide Mass Fingerprint database was employed to evaluate the obtained data. Primers were designed based on the homologous sequence in the database, and the PCL-degrading enzyme gene fragments was amplified by PCR using the genome of *Pseudomonas* sp. DS0801 as the template. The three-dimensional spatial structure was constructed using the SWISS-MODEL server.

#### Enzymatic degradation of PCL film

The PCL film was prepared by hot pressing. PCL granules were melted at 100°C using a hot press apparatus, after which followed by rapid cooling to room temperature with a cold press apparatus. A total of 1 mL of 0.2 mg/mL PCL-degrading enzyme solution was incubated with PCL films measuring 10 × 10 × 0.2 mm. The enzyme solution was refreshed daily, and the films were removed at regular intervals for thorough vacuum drying. The weights of the films were subsequently recorded to generate a weight loss curve.^10^

#### Scanning electron microscope (SEM) analysis

The morphologies of the films at various degradation stages were examined using a SEM (Hitachi, Japan). The acceleration voltage was set to 15 kV. For sample preparation, the surfaces of the films were coated with gold.

#### Quantification of monomer conversion

The monomer conversion rate was measured via an Agilent 6890 N gas chromatograph (GC). The column oven temperature was initially set to 100°C for 3 min, followed by a temperature ramp of 25°C per min until it reached 200°C, which was then held constant for 15 min. The injection port and detector temperatures were adjusted to 200°C and 250°C. Monomer conversion was assessed using butyl acetate as the internal standard and high-purity nitrogen gas as the carrier.^34^ The heat-inactivated enzyme was used as the negative control.

The monomer conversion rate (m_i_%) is calculated using the following formula^35^:

$$m_{i}\%=1-\frac{m_{s}}{m}\times \frac{f_{i}A_{i}}{f_{s}A_{s}}\times 100\%$$

m_s_, m: weight of butyl acetate and sample (mg); A_s_, A_i_: the peak area of butyl acetate and sample; f_s_, f_i_: correction factor for the relative weight of butyl acetate and sample.

#### Enzymatic polymerization and polycondensation

A total of 25 mg of purified enzyme was dried and kept into a pre-dried container holding 400 μL of ε-caprolactone. An additional 500 μL of hexane was then added. The reaction mixture was incubated at 45°C for 24 h and subsequently terminated by adding dichloromethane.^35^ The conversion rate of the ring-opening polymerization of the lactone was determined by GC (refer to method 2.12). The polycondensation ability catalyzed by PCLase0801 was investigated by replacing ε-caprolactone with 1 mM diol and 1 mM diacid, keeping other conditions unchanged.

#### Determination of molecular weight and polydispersity index (PDI)

Gel permeation chromatography (GPC) was employed to analyze the molecular weight and PDI of the products.^34^ The analysis was performed using a Waters HPLC system with tetrahydrofuran as the eluent. The elution rate was set to 1.0 mL/min. The sample was dissolved in tetrahydrofuran, and before injection, it was filtered using a 0.22 μm organic filter membrane. The injection volume was 20 μL. The calibration curve was constructed using polystyrene with standard molecular weight.

#### Nuclear magnetic resonance (NMR) test

Nuclear magnetic resonance (NMR) spectroscopy was conducted to investigate the structural characteristics of the sample. The ^1^H spectra were recorded in chloroform-d using a Bruker-400 spectrometer. Tetramethylsilane (TMS) was used as the internal standard at room temperature. The resulting spectra were analyzed using MestRec software.

#### Fourier transform infrared spectroscopy (FT-IR) analysis

The sample was analyzed by Fourier transform infrared (FT-IR) spectroscopy using a Bruker Vertex 70 instrument. The FT-IR spectrum was obtained using the standard KBr pellet method, spanning a range of 4000–400 cm^−1^, to identify characteristic absorption peaks.

#### Differential scanning calorimetry (DSC) analysis

The thermal properties of the PCL films were assessed using differential scanning calorimetry (DSC) (DSC-7C, PerkinElmer, USA). A sample weighing 6 mg was placed in a small crucible, and the analysis was conducted under a nitrogen flow, with an indium standard sample used for calibration. The analysis was performed over a temperature range of 20°C–80°C, with a heating rate of 10°C per min. Standard enthalpy values of PCL (ΔH_m_ = 139.5 J/g) were used to determine the crystallinity of PCL films, as described by Ma et al.^36^

#### Thermogravimetric analysis (TGA)

The thermal degradation properties of the product were evaluated using a TGA7 thermogravimetric analyzer (PerkinElmer, USA). Approximately 8 mg of the sample was heated from 20°C to 600°C at a rate of 10°C per min in nitrogen gas.

#### Polarizing optical microscopy (POM)

A cover glass was cleaned with alcohol and placed on a heater set to 60°C. Using tweezers, a small amount of PCL was carefully placed at the center of the cover glass. Once the PCL melted, a second cover glass was placed on top. The molten PCL was evenly compressed with tweezers until it formed a uniform sheet approximately 1 mm thick. The PCL sheet was gradually heated from ambient temperature to 90°C at a rate of 30°C per min. After a 3-min equilibration period, the temperature was decreased to 40°C. The isothermal crystallization process was conducted for 15 min, during which images of crystal morphology were captured. The same procedure was applied to commercial PCL granules (CAPA6800), which served as a control.

### Quantification and statistical analysis

Origin 2021 was used to perform Figures 3, 5, and S1–S6. All data represent the results of three independent experiments, expressed as the mean ± standard deviation (SD). One-way analysis of variance (ANOVA) was performed to determine significant differences among the groups. A *p*-value of ≤0.05 was considered statistically significant.
