# Supplementary material for: Degradation and ring-opening polymerization of poly(ε-caprolactone) by a novel enzyme from Pseudomonas sp. DS0801
Source: iScience. 2025 Nov 21;28(12):114173. doi: 10.1016/j.isci.2025.114173 (PMC12741466; doi:10.1016/j.isci.2025.114173)
Supplement: Document S1. Figures S1–S7 and Tables S1 and S2 [file mmc1.pdf]

iScience, Volume *28*

Supplemental information

Degradation and ring-opening polymerization of poly(ε-caprolactone) by a novel enzyme from *Pseudomonas* sp. DS0801

Yao Di, Wenfei Luan, Lihua Sun, Jing Qi, Hongmei Xia, Fan Li, and Niu Zhai

Fig. S1 Effect of different substrates on the lipase activity.

Fig. S2 The influence of PCLase0801 amount for ring-opening polymerization.

Fig. S3 The influence of reaction time for ring-opening polymerization of PCLase0801.

Fig. S4 The influence of temperature for ring-opening polymerization of PCLase0801.

Fig. S5 The influence of solvent system for ring-opening polymerization of PCLase0801.

Fig. S6 The influence of water activity for ring-opening polymerization of PCLase0801.

Fig. S7 Poly(ε-caprolactone) synthesized by PCLase0801.

Table S1 The influence of reaction time on M_n, M_w and PDI value of the product.

Table S2 The influence of solvent system on M_n, M_w and PDI value of the product.

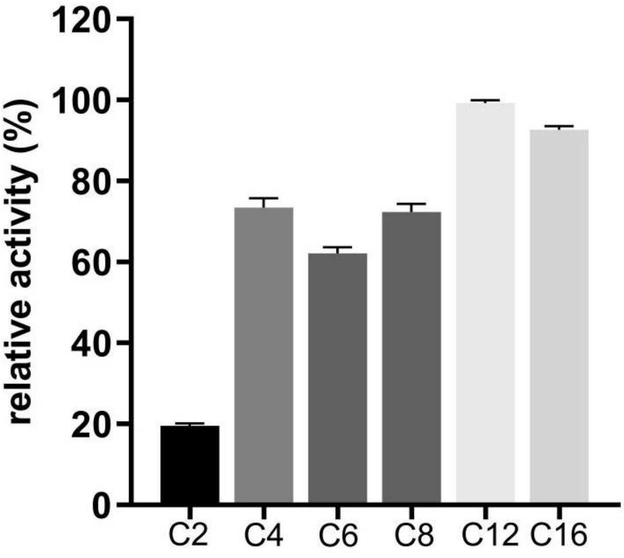

Fig.S1 Effect of different substrates on the lipase activity.

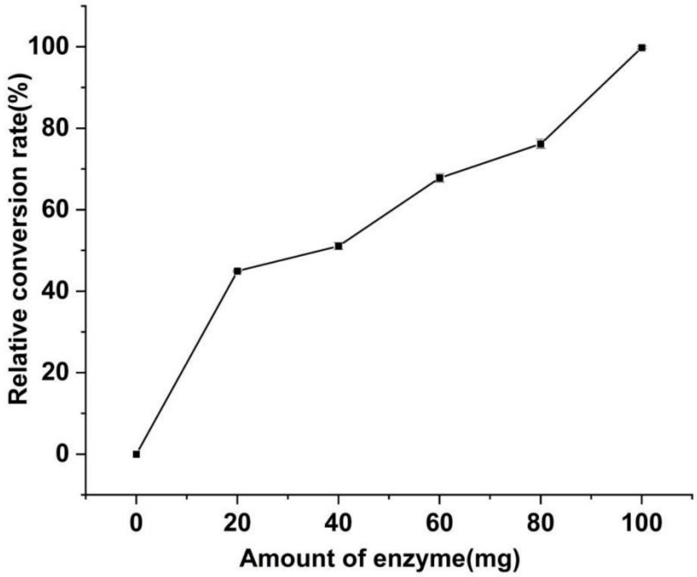

Fig.S2 The influence of PCLase0801 amount for ring-opening polymerization.

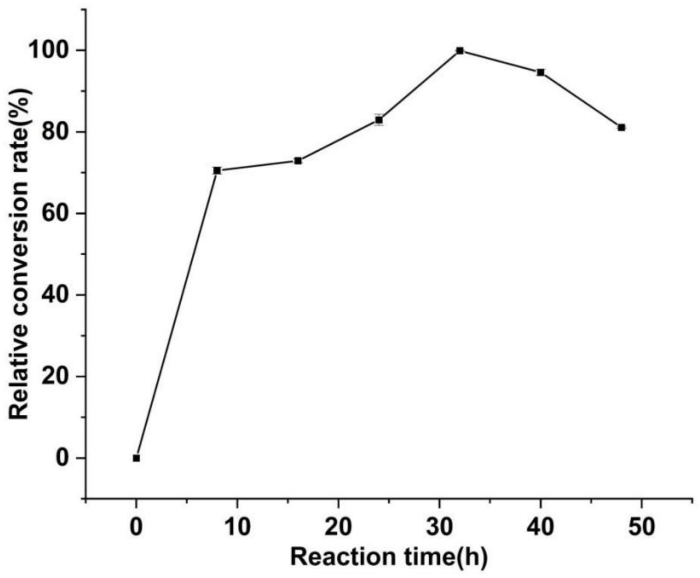

Fig.S3 The influence of reaction time for ring-opening polymerization of PCLase0801.

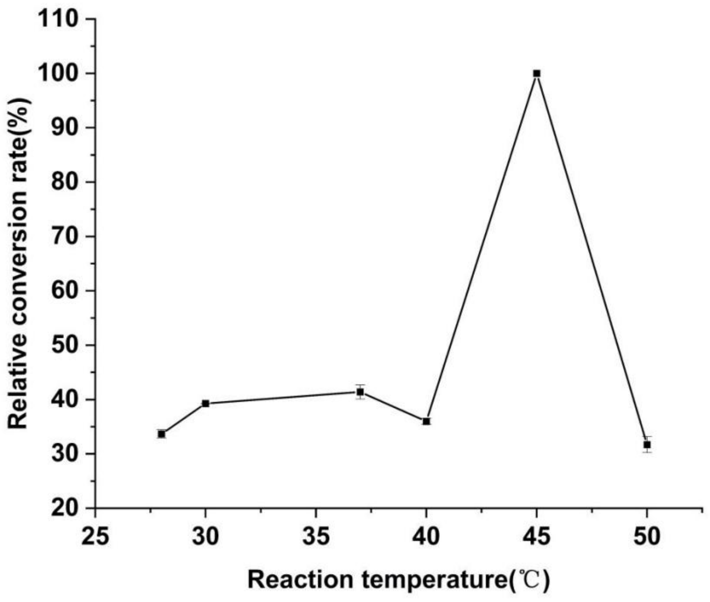

Fig.S4 The influence of temperature for ring-opening polymerization of PCLase0801.

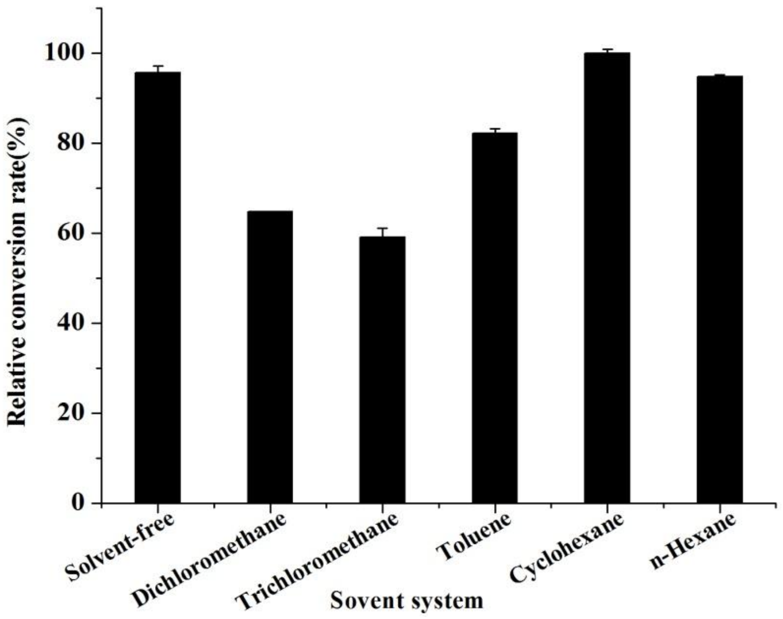

Fig.S5 The influence of solvent system for ring-opening polymerization of PCLase0801.

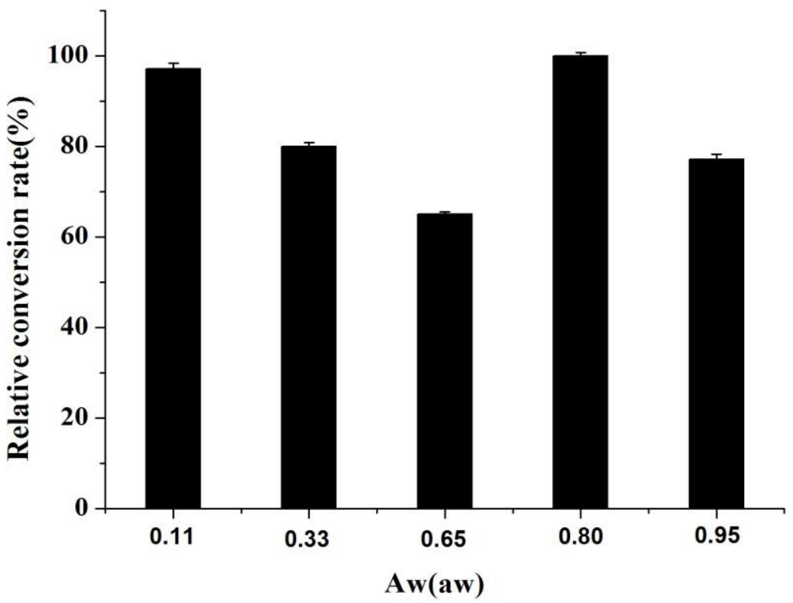

Fig.S6 The influence of water activity for ring-opening polymerization of PCLase0801.

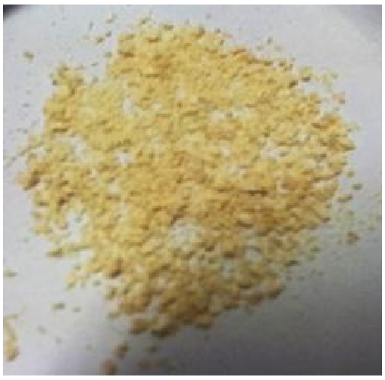

Fig.S7 Poly(ε-caprolactone) synthesized by PCLase0801.

Table S1 The influence of reaction time on Mn, Mw and PDI value of the product.

Reaction time (h)	Mn (g/mol)	Mw (g/mol)	PDI
8	4,397	5,514	1.2540
32	4,493	5,373	1.1959

Mn: number-average molecular weight; Mw: weight-average molecular weight; PDI: polydispersity; PDI= Mw/Mn.

The relative conversion rate increased slowly between 8 h and 32 h (Figure S3). The molecular weights (Mw) of the products at 8 h and 32 h were comparable, while the product obtained at 8 h exhibited a lower polydispersity index (PDI). Although the number-average molecular weight (Mn) at 32 h was slightly higher than that at 8 h, this difference had little impact on practical applications. Thus, the optimal reaction time for the PCL synthesis system was set at 8 h.

Table S2 The influence of solvent system on M_n, M_w and PDI value of the product.

Solvent	M_n(g/mol)	M_w(g/mol)	PDI
Toluene	5,317	7,516	1.4136
n-Hexane	6,050	11,074	1.7024
Cyclohexane	3,050	3,747	1.2285
Solvent-free	4,180	4,625	1.1065

Mn: number-average molecular weight; Mw: weight-average molecular weight; PDI: polydispersity; PDI= M_w/M_n.

The results (Fig. S5) showed that the relative conversion rate was higher when toluene, n-hexane, and cyclohexane were used as solvents. Among these, the product synthesized in the n-hexane system exhibited the highest molecular weight (Mw).
